# Feasibility of [^18^F]fluoropivalate hybrid PET/MRI for imaging lower and higher grade glioma: a prospective first-in-patient pilot study

**DOI:** 10.1007/s00259-023-06330-0

**Published:** 2023-07-25

**Authors:** Shahriar Islam, Marianna Inglese, Matthew Grech-Sollars, Preetha Aravind, Suraiya Dubash, Tara D. Barwick, Kevin O’Neill, James Wang, Azeem Saleem, James O’Callaghan, Giulio Anchini, Matthew Williams, Adam Waldman, Eric O. Aboagye

**Affiliations:** 1 Department of Surgery and Cancer, Faculty of Medicine, Imperial College London, Hammersmith Hospital Campus, Du Cane Road, London, W12 0NN UK; 2 Department of Brain Sciences, Faculty of Medicine, Imperial College London, Hammersmith Hospital Campus, Du Cane Road, London, W12 0NN UK; 3 Invicro Limited, Burlington Danes Building, Imperial College London, Hammersmith Hospital Campus, Du Cane Road, London, W12 0NN UK; 4 Present Address: Hull York Medical School, University of Hull, Cottingham Road, Hull, HU6 7RX UK; 5 Centre for Clinical Brain Sciences, University of Edinburgh, 49 Little France Crescent, Edinburgh, EH16 4SB UK

**Keywords:** [^18^F]fluoropivalate, FPIA, PET/MRI, Glioma, Short-chain fatty acid

## Abstract

**Purpose:**

MRI and PET are used in neuro-oncology for the detection and characterisation of lesions for malignancy to target surgical biopsy and to plan surgical resections or stereotactic radiosurgery. The critical role of short-chain fatty acids (SCFAs) in brain tumour biology has come to the forefront. The non-metabolised SCFA radiotracer, [^18^F]fluoropivalate (FPIA), shows low background signal in most tissues except eliminating organs and has appropriate human dosimetry. Tumour uptake of the radiotracer is, however, unknown. We investigated the uptake characteristics of FPIA in this pilot PET/MRI study.

**Methods:**

Ten adult glioma subjects were identified based on radiological features using standard-of-care MRI prior to any surgical intervention, with subsequent histopathological confirmation of glioma subtype and grade (lower-grade – LGG – and higher-grade – HGG – patients). FPIA was injected as an intravenous bolus injection (range 342–368 MBq), and dynamic PET and MRI data were acquired simultaneously over 66 min.

**Results:**

All patients tolerated the PET/MRI protocol. Three patients were reclassified following resection and histology. Tumour maximum standardised uptake value (SUV_max,60_) increased in the order LGG (WHO grade 2) < HGG (WHO grade 3) < HGG (WHO grade 4). The net irreversible solute transfer, Ki, and influx rate constant, K1, were significantly higher in HGG (*p* < 0.05). Of the MRI variables studied, DCE-MRI-derived extravascular-and-extracellular volume fraction (v_e_) was high in tumours of WHO grade 4 compared with other grades (*p* < 0.05). SLC25A20 protein expression was higher in HGG compared with LGG.

**Conclusion:**

Tumoural FPIA PET uptake is higher in HGG compared to LGG. This study supports further investigation of FPIA PET/MRI for brain tumour imaging in a larger patient population.

**Clinical trial registration:**

Clinicaltrials.gov, NCT04097535.

**Supplementary information:**

The online version contains supplementary material available at 10.1007/s00259-023-06330-0.

## Introduction

Glioma is the most common primary malignant brain tumour in adults, and accurate malignancy characterisation is important to optimise treatment pathways, as survival can range from 10 to 20 years in the case of IDH mutated, 1p/19q co-deleted lower-grade glioma (LGG) to 15 months for IDH wild-type glioblastoma, the most aggressive higher-grade glioma (HGG) [1–4]. Conventional contrast-enhanced MR imaging (CE-MRI) is routinely used in the initial evaluation of brain tumours but has a limited role in discriminating LGG from HGG; tissue confirmation of the diagnosisis, therefore, important in patients suspected of harbouring a primary glial neoplasm. Other MRI methods [5] that quantify perfusion, blood volume, diffusion and tumour metabolite levels provide improved discrimination of malignancy [4, 6] as well as determining recurrence and therapy response [7, 8]. Several PET radiotracers have also been tested in the setting of brain tumour detection [9]. The most widely used of these are amino acid tracers like [^18^F]fluoroethyl-L-tyrosine, yet for these, conventional static imaging has limited application [10, 11]; dynamic imaging improves lesion characterisation [12–15]. Newer amino acid tracers such as [^18^F]fluciclovine offer static imaging capabilities [16]. Hybrid PET/MRI, having overcome numerous technical challenges, including the efficient performance of PET within a magnetic field and use of MR-based attenuation correction [17], now provides the opportunity to combine novel PET and multi-parametric MRI for single-visit pre-surgical brain tumour characterisation [18]. The benefit of MRI in PET/MRI investigations has been incremental and has rarely made full use of either multi-modal MRI or kinetic co-analysis, and a saltatory approach would require simultaneous investigation of detailed multi-modal multi-parametric PET/MRI data.

The critical role of short-chain fatty acids (SCFAs) in brain tumour biology has come to the forefront. In particular, the report by Machimo and co-workers [19] that brain tumours have the capacity to oxidise acetate more so than glucose (but not glutamine) indicates a unique adaptation within the brain niche. The authors further assert that tumour grade-correlated ACSS2 is a key enzyme required for this metabolic vulnerability and potential therapeutic target of tumours. The ability of acetate to characterise brain tumours has been exploited in PET with [^11^C]acetate [20], a radiotracer that lacks widespread clinical application. We recently reported the discovery of a non-metabolised fluorine-18 radiolabelled compound for imaging SCFA transcellular flux [21–23]. The radiotracer, [^18^F]fluoropivalate (FPIA), shows low background activity in most organs except eliminating organs – liver and bladder – and has appropriate dosimetry for routine clinical use [24]. In the present pilot study, we assess multi-parametric multi-modal dynamic FPIA PET/MRI to determine if this approach provides discrimination of glioma grade.

## Materials and methods

### Patients

Ten patients underwent detailed multi-parametric multi-modal dynamic FPIA PET/MRI. All patients were identified through a dedicated neuro-oncology multi-disciplinary meeting based on radiological features using standard-of-care MRI prior to any surgical intervention (either surgical biopsy or debulking); following surgical intervention, all subjects had histopathological and molecular confirmation of glioma subtype and grade and reclassified as LGG or HGG, if necessary. Inclusion criteria were patients > 18 years, lesions at least 1 cm in minimum diameter on MRI, WHO performance status 0–2 and estimated glomerular filtration rate > 60 within 3 months of scan date. All female patients of childbearing age were required to have a negative pregnancy on the day of the scan to be included. The exclusion criteria comprised any chronic illness or musculoskeletal condition that would prevent the patient from completing the study, concurrent therapy with any other investigational agent within 14 days of the scan date and any contraindication to MRI.

### Radiopharmaceutical preparation

FPIA radiosynthesis was carried out using a GE Fastlab™ automated radiosynthesis platform to produce GMP-grade radiopharmaceutical. The automated radiosynthesis technique has been previously described in detail by Dubash et al. [24]. Briefly, the precursor, methyl 2,2-dimethyl-3-[(4-methylbenzenesulfonyl)oxy]propanoate, was radiolabelled by displacement of the tosylate group with [^18^F]fluoride to produce the methyl ester of FPIA. This compound was then hydrolyzed under basic conditions to give FPIA, which was purified by semi-preparative HPLC using biocompatible solvents (15% EtOH, 85% sodium dihydrogen phosphate buffer, pH 4.5). The fraction containing FPIA was diluted in water and passed through a sterile filter into a sterile vile for clinical use. The chemical and radiochemical purities of the final product were determined by HPLC. A range of quality control tests were performed according to European Pharmacopoeia [24].

### Image acquisition

All subjects underwent dynamic pre-treatment FPIA PET/MRI on a Signa™ 3.0 T scanner (GE Healthcare Systems, USA) in a single-bed position using a 3.0 T GEM HNU coil. Before FPIA was injected, 3-plane MRI localisation was performed, and the PET field of view was defined (centred on the superior margin of the thalamus to include the entire brain). FPIA was injected as an intravenous bolus injection (range 342–368 MBq; mean 347.4 MBq), and PET data were acquired in list-mode format (0–66 min) and reconstructed using VUE point FX (VPFX; 3D OSEM plus time of flight reconstruction) at 192 × 192 matrix size, 2 iterations and 28 subsets with a 5-mm Gaussian filter (no Z filter) into the following frame × duration: 10 × 15 s, 3 × 60 s, 5 × 120 s, 9 × 300 s and 1 × 360 s (66.5 min). Simultaneous MRI brain acquisition (commencing after 10 min following FPIA injection) included MR-based attenuation correction (MRAC) – Zero Echo Time pulse sequence (ZTE) – together with structural and functional MRI sequences including pre- and post-contrast T_1_ volume, fluid-attenuated inversion recovery (FLAIR) volume and diffusion-weighted imaging (DWI). Three perfusion sequences were acquired: pulsed arterial spin labelling (pASL), dynamic contrast-enhanced MRI (DCE-MRI) and dynamic susceptibility-MRI (DSC-MRI). As a result of performing both DCE-MRI and DSC-MRI, two boluses of Gadovist were required (1.0 mmol/mL Gadovist solution at 0.1 mmol/kg subject body weight, injected at 3 mL/s, followed by immediate 20 mL saline flush at 3 mL/s). A single venous blood sample of 5 mL for venous carnitine was obtained in patients prior to imaging. The first 5 patients enrolled in the study also had continuous arterial blood sampling acquired from the start of scanning for 10 min together with discrete arterial blood samples taken at 2.5, 15, 30, 45 and 60 post-injection. All discrete samples were analysed for counts (cross-calibrated with continuous arterial sampling) and metabolites. For the second cohort of patients who did not have arterial blood sampling, a population average input blood was derived such that all patients had a metabolite-corrected input function.

### Image analysis

Regions of interest used for quantification were identified and manually segmented by a neuroradiologist with over 10 years of experience (SI). Two sets of volumes of interest (VOIs) were generated per subject using the volumetric FLAIR and volumetric T_1_ post-contrast sequences, although all MRI sequences including T_2_ and pre-contrast T_1_ were reviewed to ensure an accurate assessment of the lesions. VOIs drawn on volumetric FLAIR sequences included all solid tumour components and perilesional abnormal FLAIR signal regions. Areas of necrosis were eliminated from the final MRI VOI. On the volumetric T_1_ post-contrast VOIs, enhancing and non-enhancing solid tumour components were segmented, again excluding any necrotic fluid components. For each lesion, VOI, a contralateral VOI of the same volume in a radiologically healthy brain, was segmented. The superior sagittal sinus was segmented for each subject to allow for whole-blood correction. All segmentations were performed using freely available segmentation software ‘ITK-SNAP’ (http://www.itksnap.org/pmwiki/ pmwiki.php). The last frame of the PET image was used for (static) visualisation of uptake. To determine image overlap, additional SUV masks – SUV_30_ and SUV_40_ – were derived. We employed an adaptation of the contour-based segmentation by Besson and co-workers within ITK-SNAP software by finding the SUV_max_ and then thresholding it to SUV_30_ or SUV_40_ to SUV_max_ ([25]). Overlap was assessed by DICE scores.

### PET and MRI data quantification

The quantification of FPIA PET data involved the evaluation of standardised uptake value (SUV) variables including SUV_c_ and TBR_max_, and kinetic modelling variables including K_1_ and K_i_ derived from compartmental modelling and Patlak analysis, respectively [26]. The analysis of MRI data enabled the quantification of dynamic contrast-enhanced MRI (DCE)-contrast agent plasma/interstitium transfer rate constant (K^trans^) [27]; dynamic susceptibility contrast (DSC) MRI-cerebral blood volume corrected for leakage (CBV; CBVlc) and cerebral blood flow (DSC-CBF), together with contrast agent mean transit time (MTT) and time to peak (TTP) [28–30]; non-contrast-based arterial spin labelling MRI-cerebral blood flow (ASL-CBF) [31, 32]; and diffusion-weighted imaging MRI-apparent diffusion coefficient (DWI-ADC) [33]. To allow the comparison with DSC-MRI-related perfusion parameters, both ASL-CBF and DSC-CBF, CBV, CBVlc, MTT and TTP have been normalised to contralateral white matter [31]. Spectral analysis was used to determine retention dynamics beyond perfusion [34].

### Carnitine levels

Blood samples were taken at baseline prior to scanning for measuring non-esterified fatty acids (NEFA) and carnitine. All samples were centrifuged (1942 g, room temp, 5 min) within 30 min of collection and stored at − 80 °C until transfer to laboratories for analysis. Analysis was performed as previously reported [24].

### Enzymology and survival in archival tissues

Formalin-fixed, paraffin-embedded LGGs (*n* = 7) and HGGs (*n* = 22) were obtained from our tissue bank (R18019-1A; ICHTB HTA licence: 12,275 Research Ethics Committee Wales approval: 17/WA/0161) and sectioned for immunohistochemistry. We evaluated the expression of Ki-67, ACSS1/2, ACSS2, ACSS3, organic cation transporter/sodium-dependent high-affinity carnitine transporter solute carrier family 22 member 5 (SLC22A5) and carnitine-acylcarnitine translocase solute carrier family 25 member 20 (SLC25A20). Briefly, after antigen retrieval, sections were incubated with primary antibodies (mouse monoclonal MIB-1 Ki-67, 1:200; rabbit polyclonal AceCSI ACSS1/2, 1:50; rabbit polyclonal ACSS2, 1:50; rabbit polyclonal ACSS3, 1:50; rabbit polyclonal SLC22A5, 1:300; and rabbit polyclonal SLC25A20, 1:1500; Abcam). Following incubation with secondary antibodies, samples were counterstained with haematoxylin and mounted for analysis. Samples were scored based on intensity and coverage (0–300) by an experienced pathologist (FM).

### Statistical analysis

Statistical analysis was performed using GraphPad Prism version 7. Summary data are reported as mean ± SD. We did not assume normality due to only *N* = 10 patients being assessed; the nonparametric Wilcoxon test was used to assess any statistically significant differences. *P*-value ≤ 0.05 was considered significant. A penalised least squares classification analysis method that performs both variable selection and regularisation least absolute shrinkage and selection operator, LASSO [35], was used to select the combination of MRI and PET variables likely to discriminate grade. The web server, Gene Expression Profiling Interactive Analysis (GEPIA, http://gepia.cancer.pku.cn/) database, was used to verify the overall survival conferred by ACSS2 or SLC25A20 in a combined LGG and HGG dataset from TCGA. Cox proportional hazard model was used; the hazard ratio was determined with Kaplan–Meier curves dichotomised by the median. The *P*-value was determined by the log-rank test.

## Results

### Patient demographics

Table 1 shows the patient characteristics for the study. In total, 10 treatment naïve patients who completed all standard-of-care MRI, research PET/MRI and histopathology were included in the analysis; of the total 11 patients, patient 6 did not undertake any of the PET/MRI and was excluded from further analysis. Three patients, originally LGG, progressed or were reclassified as HGG following histopathology assessment. The confirmed histopathology and WHO grades (patient) are indicated in Table 1 and include two WHO grade II LGG (IDH mutant; 1 with and 1 without 1p/19q del), three WHO grade III HGG (IDH mutant; 1 without and 2 with 1p/19q del) and five WHO grade IV HGG (IDH wild-type glioblastoma, GBM) [2].

**Table 1:** Clinical characteristics

Patient	P01	P02	P03	P04	P05	P07	P08	P09	P10	P11
Age	31	78	71	58	54	39	68	73	63	60
Sex	M	M	M	F	M	F	M	F	M	M
Ethnicity	White	White	White	White	Black	Asian	Other	Asian	Black	White
Treatment type	Surgery LG	Surgery HG	Surveillance LG, biopsy on progression†	Surgery LG	Surgery LG†	Surveil-lance LG; Surgery†	Surgery HG	Surgery HG	Surgery HG	Surveil-lance LG; Surgery
	Elective debulk	Elective debulk	Biopsy on transformation; chemoradiotherapy	Elective debulk	Biopsy; 1 cycle chemotherapy	Debulk; chemoradiotherapy	Debulk; chemoradiotherapy	Debulk	Debulk; chemoradiotherapy	Complete debulk; chemoradiotherapy
Time (days) between FPIA and surgery	33	4	107	1	16	101	10	11	2	75
Number of samples taken for histology	8	3	2	3	2	2	3	2	2	2
Histopathology (including % expression in neoplastic cells)	Diffuse astrocytic, Ki67 4%, p53 20%	Astrocytic, Ki67 15–20%, p53 > 20%	Diffuse astrocytic, Ki67 5%, p53 widespread expression	Diffuse glioma, Ki67 15–20%	Diffuse astrocytic, Ki67 2%, focal areas of early transformation	Oligodendroglioma, Ki67 8%, p53 5%	Cellular glial tumour with astrocytic morphology, Ki67 15%, p53 overexpressed > 20%	Astrocytic, primitive neuronal components, Ki67 > 50%, p53 diffuse expressed	Cellular glial tumour with astrocytic morphology, Ki67 15%, p53 focal overexpressed	Oligodendroglioma, Ki67 10%, p53 not overexpressed
WHO grade histology	II LGG astrocytoma	IV HG GBM	III astrocytoma	IV HG GBM	II L–focal areas of early transformation	Mixed II and III	IV HG GBM	IV HG GBM	IV HG GBM	II LGG oligodendroglioma
WHO grade patient	II LGG	IV HGG	III astrocytoma	IV HGG	III astrocytoma	III oligodendroglioma	IV HGG	IV HGG	IV HGG	II oligodendroglioma
Routine CE-MRI (CE-T1 MRI and T2 FLAIR) for initial staging	LGG	HGG	LGG but transformed on surveillance – HGG	HGG	HGG	LGG	HGG	HGG	HGG	LGG
Molecular phenotype	IDH1mu; ATRX loss; p53mu, 1p/19q aneuploidy	IDHwt; MGMT-methylated; ATRXwt, p53wt	IDH1mu; ATRX loss; p53 mu	IDH1wt; MGMT unmethylated; p53wt;; EGFR-VIIImu, 1p/19q aneuploidy	IDH1mu; ATRXmu; p53mu, 1p/19q aneuploidy; 1p/19q del in an aneuploid clone	IDH1mu; ATRX loss; p53wt, 1p/19q del	IDH1wt; ATRwt;; p53mu, MGMT unmethylated; TERTmu; pTENmu; CDK4 amplification	IDH1wt; ATRXwt,; p53 mu, MGMT methylated > 25%; TERTmu; pTENmut	IDH1/2wt; p53wt; MGMT methylation low < 5%, TERT mu;	IDH1mu; ATRXwt; p53wt; 1p/19q del; TERT mu;
Overall survival (months)	64	1.6	61.2	7.05	42	29.03	5.5	3	13.7	25

^†^Progressed or reclassified – originally surveillance for LGG

*LGG*, lower-grade glioma; *HGG*, higher-grade glioma

### FPIA lesion uptake associates with grade

**Fig. 1:**
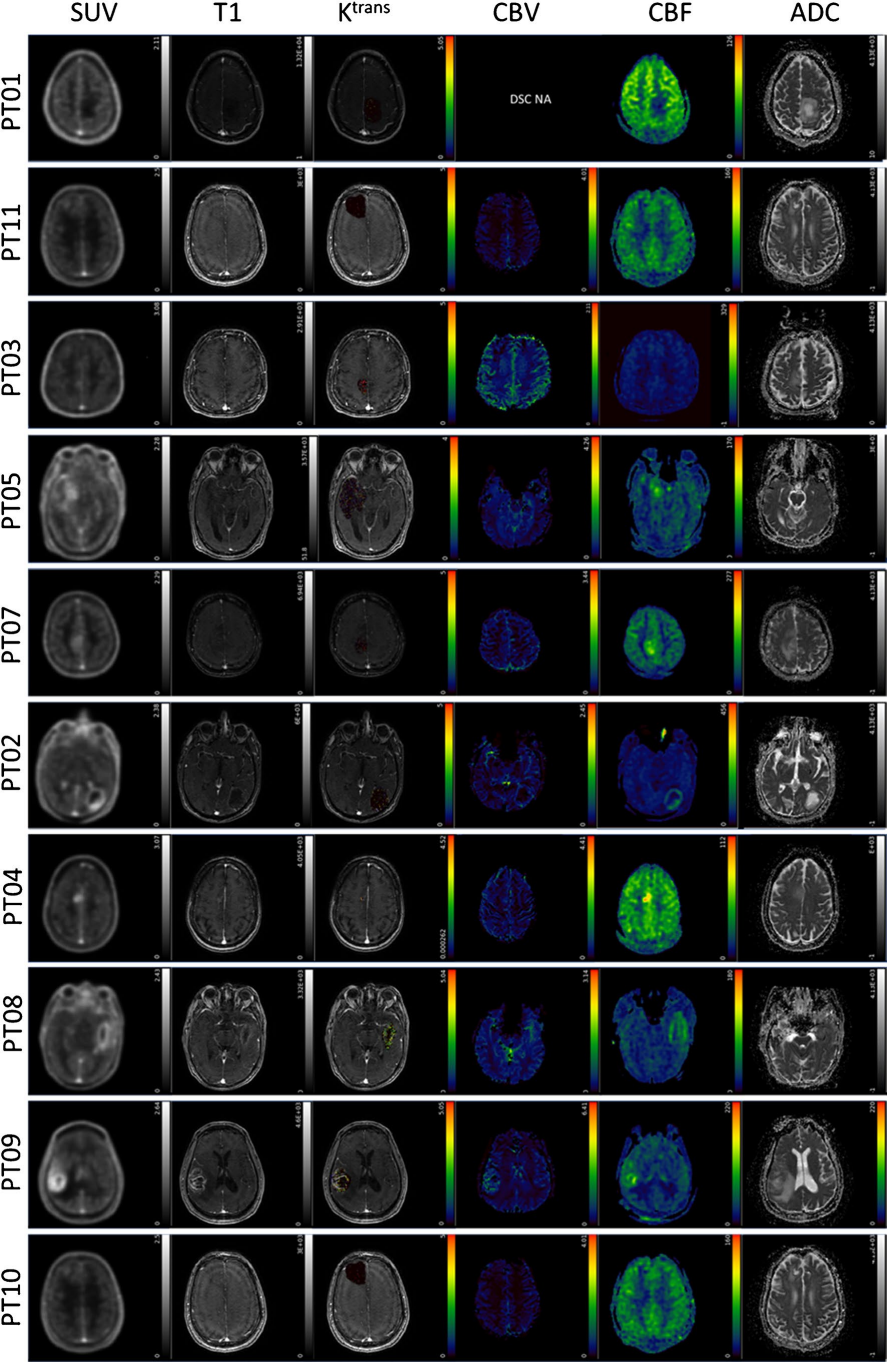
FPIA PET/MRI images in patients with grades II, III and IV gliomas. Axial PET and MRI images for grade II (PT01 and PT11), grade III (PT03, PT05, PT07) and grade IV (PT02, PT04, PT08, PT09, PT10) glioma patients including standardised uptake value for the last 5 time frames (SUV) from FPIA PET, T1 weighted MRI sequence, dynamic contrast-enhanced MRI (DCE)-contrast agent plasma/interstitium transfer rate constant (Ktrans), dynamic susceptibility contrast (DSC) MRI-cerebral blood volume (CBV), arterial spin labelling MRI-cerebral blood flow (CBF) and diffusion-weighted imaging MRI-apparent diffusion coefficient (ADC)

**Fig. 2:**
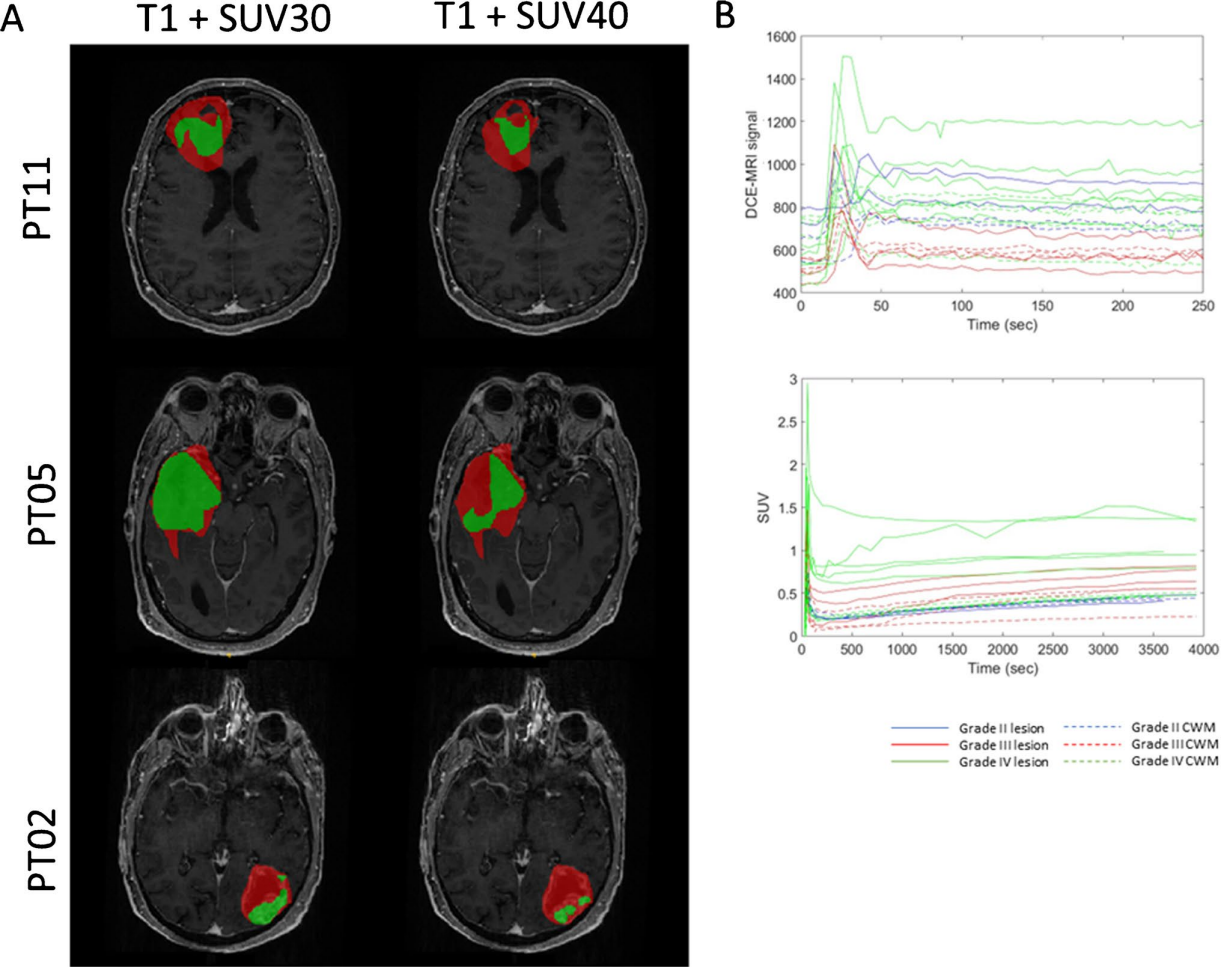
Image congruence. (**A**) Lesion mask outlined on the post-contrast T1 MRI image (red) and on the SUV_40_ and SUV_30_ (green). (**B**) Dynamic contrast-enhanced (DCE) MRI and SUV average tissue time activity curves in grade II (blue), grade III (red) and grade IV (green) for lesion or CWM

All patients tolerated the PET/MRI protocol. Patient 1 did not have DSC perfusion sequences due to equipment failure. T1 MRI maps were used for quantification of PET data. Tumour lesions were visible above background contralateral brain parenchyma in all HGGs, but less so in LGG (Fig. 1; Supplementary Fig. S1). Contrast, depicted quantitatively by tumour SUV_max_/contralateral brain SUV_mean_ ratio at 60 min (TBR_60_max_) was found to be ≥ 2 in all tumours. This indicates that FPIA is heterogeneously taken up above background healthy brain in both LGGs and HGGs. A review of all PET image slices showed blood pool FPIA localisation in some images. We implemented a blood volume correction to improve image contrast based on the subtraction of normalised blood volume within the VOI of the sagittal sinus. Lesions in high blood volume brain regions showed visual improvements, with no remarkable improvements seen in other lesions (Supplementary Fig. S2). Comparison of uncorrected and blood volume-corrected standardised uptake value averaged over the last 5 frames of acquisition (SUV_mean_ of last 26 min; unitless) showed enhanced visual contrast for some lesions (Supplementary Fig. S2) indicating that lesion visualisation could be improved with such correction. Since the optimal time for FPIA PET was not known, a dynamic scan was conducted over 66.5 min. When SUV_30_ and SUV_40_ were determined, these volumes did not overlap with T1 MRI volumes; DCE-MRI curves did not overlap with PET uptake curves either (Fig. 2). Based on the increasing time versus radioactivity curves (Fig. 2), 60 min mid-time frame was selected for static PET analysis. Data are presented in Supplementary Table S1. Maximum voxel FPIA uptake (SUV_60_max_) was found to increase with grade in the order LGG (WHO grade II; range 0.8974–0.9108) < HGG (WHO grade III; range 1.1520–1.5910) < HGG (WHO grade IV; range 1.6564–2.6358). Supplementary Table S1 also shows TBR_60max_.

**Fig. 3:**
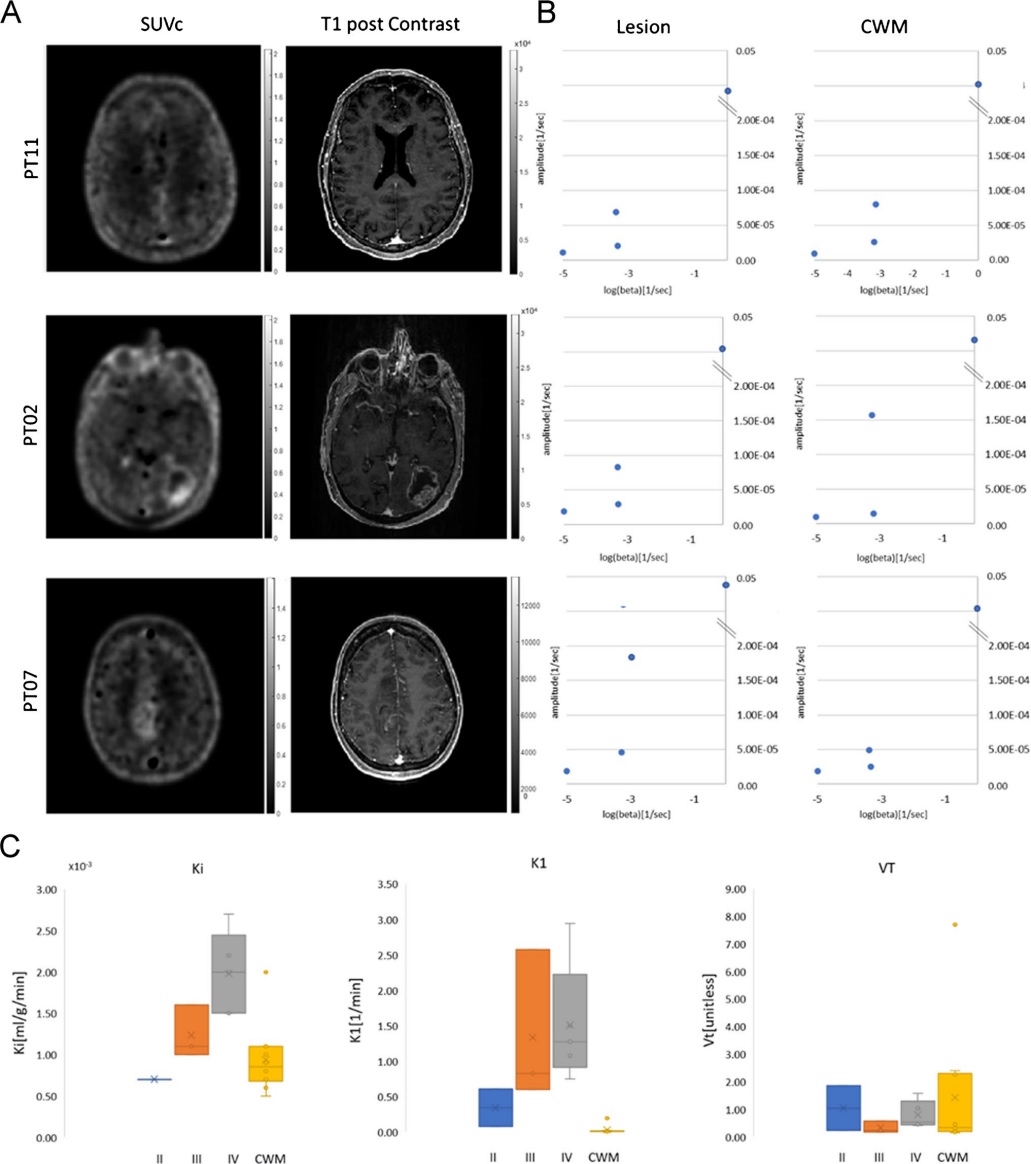
Blood to brain and tumour transit of FPIA. (**A**) Comparison of structural (longitudinal relaxation time on MRI-T1 post-contrast) and functional (FPIA PET SUVc) image sets from grade II (PT11), grade III (PT02) and grade IV (PT07) patients. (**B**) Spectrum of kinetic components evaluated in the lesion and CWM by spectral analysis. (**C**) Boxplots showing K1, Ki and V_T_ parameters in grades II, III, IV and CWM

Using spectral analysis, slow components were found for CWM and different tumour lesions (Fig. 3A–C; Supplementary Fig. S3). Values for the steady-state net influx rate constant (Ki) were non-zero (Fig. 3C), indicating steady-state net transfer of FPIA from the blood into tumour tissue for all lesions (and healthy brain); Ki was more pronounced in the GBMs and increased in a grade-dependent manner.

### PET and MRI methods emphasise tumour lesions to different extents

For HGG, tracer uptake was apparent to visual inspection on FPIA PET and MRI (Fig. 1). Discordance between FPIA PET and MRI maps was also evident (Fig. 1B) (for consistency in Supplementary Table S1, we analysed VOIs defined on T1). A summary of concordance/discordance – DICE scores and volume overlap – in all patients is presented in Table 2. Figures 2 and 3 show that FPIA uptake is not simply the result of BBB disruption and, in terms of grade relationship, is not necessarily related to ADC or CBF (Supplementary Fig. S4).

**Table 2:** Results of the comparison between the mask outlined over the post-contrast-enhanced T1-weighted MRI sequence and SUV40 and SUV30

	DICE		% Volume variation	
	MRI vs. SUV40	MRI vs. SUV30	MRI vs. SUV40	MRI vs. SUV30
PT01	0.1190	0.1370	1288%	11,413%
PT02	0.1794	0.3655	902%	319%
PT03	0.0903	0.2063	746%	345%
PT04	0.5351	0.3731	− 55%	− 75%
PT05	0.5555	0.5980	− 3%	− 29%
PT07	0.3979	0.2613	82%	181%
PT08	0.5646	0.7060	85%	20%
PT09	0.1075	0.2687	1430%	451%
PT10	0.6780	0.8057	82%	25%
PT11	0.3259	0.3993	74%	18%

Dice scores and %volume variation were evaluated as ((volume_mri – volume_suv)/volume_suv)*100

**Fig. 4:**
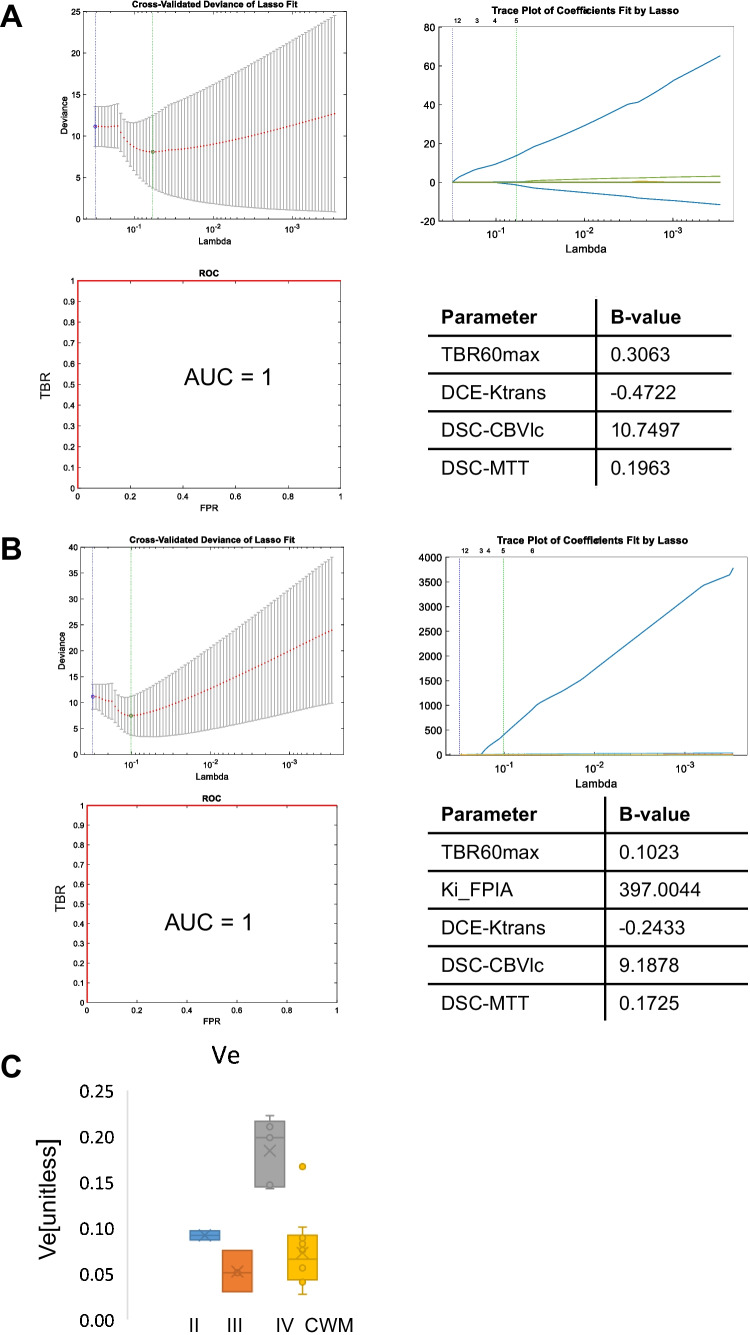
Assessment of the objective combination of PET and MRI variables for grade prediction. The least absolute shrinkage and selection operator (LASSO) was applied to extract a composite vector for tumour grade prediction from 24 (dynamic included) or 20 (static only) parameters determined by analysis of simultaneously acquired PET and MRI data. Five-fold cross-validation was performed to select lambda minimum to give the minimum cross-validated error for classifying LGG (grade II) versus HGG (grades III and IV) when (**A**) the PET variables were restricted to static data or (**B**) included dynamic PET data. The feature coefficients (b value) are indicated. (**C**) Boxplots showing v_e_ parameter in grades II, III, IV and CWM

Of the MRI variables studied, DCE-MRI-derived extravascular-and-extracellular volume fraction (v_e_) appeared to be the only discriminatory variable; high in GBM (range 0.1427–0.2225 unitless) compared to other lesions (range 0.0303–0.0968; Supplementary Table S1). No differences in quantitative variables of v_e_ were seen between LGG and HGG (WHO grade III). With the limited number of patients in this pilot study, we wondered if certain PET and MRI variables could be complementary in predicting grade LGG or HGG, a decision point for change in clinical management. The LASSO-penalised regression approach appeared to select FPIA PET and perfusion variables as the optimal combination, whether dynamic or static PET variables were provided as input (Fig. 4A,B). Of note, v_e_ (Fig. 4C) was not selected.

### Carnitine blood levels

We wanted to assess the level of variation of plasma carnitine and whether plasma carnitine levels will influence kinetic parameters (analogous to glucose in FDG studies). As a fasting protocol was not implemented, acyl, free and total carnitine levels varied markedly between patients (Fig. S5A,B; data from two samples per patient averaged). There was no relationship between carnitine levels and radiotracer uptake (SUV or Ki).

### Investigating SCFA enzymology in view of FPIA kinetics

Given the discriminatory role of K1 and Ki, we investigated transporters and enzymes involved in SCFA uptake and carnitine-related metabolic transformation [22] by immunohistochemistry in a distinct archival cohort of LGGs (*n* = 7) and HGGs (*n* = 22) from our brain tissue bank to see if these tumour types expressed different levels of these transporters or enzymes. Ki-67 expression, used to verify the phenotypic differences between LGG and HGG, had a high range of values in HGG compared to LGG, which was restricted to low values; although absolute expression levels were typical of that seen in glioma [36], the group difference did not reach statistical significance (Supplementary Fig. S6). The average values for all proteins, including SLC22A5 (OCTN2), SLC25A20 (carnitine-acylcarnitine translocase, CACT), ACSS1/2, ACSS2 and ACSS3, were higher in HGG compared to LGG but only reached significance for SLC25A20 and ACSS1/2. Of note, for ACSS1/2, we employed the same antibody used by Mashimo et al. [19] for ACSS2, but in the intervening period, reclassified as a dual ACSS1 and 2 antibody.

## Discussion

We report the first study of FPIA PET in human tumours. In this exploratory PET/MRI phenotyping study, we show that FPIA PET tumour uptake (SUV_60_max_) increases in a glioma grade-related manner and that DCE-MRI-derived extravascular-and-extracellular volume fraction (v_e_) was higher in GBM (grade IV) compared to grades II–III tumours. FPIA is a novel tracer under evaluation for brain tumours. Several lines of evidence indicate that this tracer is a candidate for imaging glioma: (a) The tracer shows high contrast and growth-related uptake in orthotopic models of glioma in mice, with contrast surpassing FDG PET [22]; (b) biodistribution of the tracer in healthy human volunteers shows low background uptake, except in eliminating organs [24]; and (c) this present study shows that the tracer has high contrast in a grade-dependent manner, with associated survival duration in our cohort: 25–64 months, 29–61 months, and 1.6 to 13.7 months, respectively, for WHO grades II, III, and IV, respectively.

The evolving understanding of the differential use of SCFAs by human brain tumours has come to the fore [19], and tracers for imaging this phenotype beyond [^11^C]acetate – which is difficult to distribute to other sites without on-site cyclotron – will enable this biology to be appreciated and form the basis of clinical diagnostic programmes for brain tumours. Ki (net solute transfer at steady state) and K1 (product of blood flow and the unidirectional first-pass extraction fraction) were higher in HGG compared to LGG, with K1 increasing in the order WHO grade II < WHO grade III < WHO grade IV. FPIA being a SCFA transports across cell membranes supported by the ubiquitous monocarboxylate transporters (MCTs) and sodium-coupled monocarboxylate transporters with several members, whose expression and functions in tissue are complex [37]. We show that mRNA expression of the prototypical MCT, SLC16A1 (MCT1), for instance, is higher in gliomas compared with corresponding normal brain tissue but otherwise non-discriminatory, while that of SLC25A20 is more emphasised in HGG (Supplementary Fig. S6). As FPIA is positively correlated with SLC25A20 protein expression (together with ACSS2, SLC22A5) [22], and our current study shows higher SLC25A20 protein expression in human HGG (grades III and IV), we suggest that SLC25A20, a mitochondrial enzyme that transports acylcarnitines from inside the cell into mitochondria, is worthy of further elaboration as an enzyme that contributes to the higher FPIA uptake in HGG. SLC25A20 studies were conducted in a separate, larger cohort to understand variations of enzymes that regulate SCFA metabolism. Future studies should examine, particularly SLC25A20, in relation to FPIA uptake. Of note, the mechanism of uptake of FPIA is distinct from that of amino acid tracers including [^18^F]fluoroethyl-L-tyrosine and [^18^F]fluciclovine [10, 11, 16]. Dynamic scans were conducted to verify the best imaging time point within 66.5 min. Based on the temporal changes in FPIA uptake in most tumours, we suggest an uptake time of 60 min (mid-time frame) for future static FPIA PET protocol. Indeed, this suggested protocol has been implemented in a new FPIA study (clinicaltrials.gov/ct2/show/NCT05801159).

CE-T1-CE MRI is the gold standard for high radiation dose boost and surgical resection, with T2/FLAIR regions considered less relevant due to oedema confounding tumour infiltration. With re-irradiation and aggressive resection becoming routine, the ability to accurately define the correct tumour target is urgent. A major consideration in evaluating new brain radiotracers is their ability to transit the blood–brain barrier (BBB). The criteria often used for asserting BBB transit are ‘visible PET uptake in the absence of MRI contrast and discordance between MRI and PET data’. These criteria depend, however, on how many such lesions exist in the cohort studied; on the basis of such criteria, [^11^C]methionine and 6-[^18^F]-fluoro-L-DOPA (FDOPA) are considered tracers that cross the BBB [38]. Another approach is to determine the rate constant for the net irreversible transit of solute from blood to tissue at a steady state (non-zero Ki) described by Patlak et al. [39, 40]. The non-zero Ki seen in CWM and all grades of brain tumours (Fig. 3C) suggests that FPIA crosses the intact BBB. This inference is supported by the high and variable partitioning of FPIA between blood and healthy brain tissue (V_T_), despite low K1, as well as the presence, in the spectral analysis output, kinetic components that are slower than simply delivery (Fig. 3B). SCFAs, charged at physiologic pH, can be transported into brain tissue by transporters including the monocarboxylate transporters, which are abundant in healthy brain tissue, blood–brain barrier and brain tumours; thus, the non-zero Ki and high V_T_ of FPIA are unsurprising [37, 41]. Expectedly, other properties of the variably compromised BBB in tumours, e.g. perfusion, may also contribute to overall SUV. The discordance between SUV and CE-MRI (Fig. 2A,B) further supports the notion that FPIA tumour uptake does not simply reflect compromised BBB. It should be noted that while the present report shows that FPIA tumour uptake correlates with tumour grade, albeit in a small cohort of patients, a consensus has not been reached for either [^11^C]methionine or FDOPA regarding correlations between PET uptake and WHO grades II, III and IV, with high FDOPA and [^11^C]methionine uptake seen in WHO grade II oligodendroglioma compared to IDH-mutant astrocytoma in some reports [42, 43].

A limitation of this study is its exploratory nature in 10 patients. Thus, any potential implied use of the technology can only form the basis of future studies. These potential applications include (a) the ability to use FPIA as a virtual biopsy of glioma malignancy to detect LGG transformation, target surgical biopsy and plan surgical resections, and (b) to exploit the tracer for target volume delineation to plan stereotactic radiosurgery. The period between imaging and histological diagnosis was variable (> 100 days in P3 and P7); furthermore, for P3, the biopsy was clinically indicated, which could have missed the most aggressive part of the tumour. These variations, which were beyond our control, are limitations of the study. The grading of glioma has been recently modified [2]. In our study, there were five IDH mutant tumours from two grade II diffuse glioma and three mixed/transforming WHO grade III HGG patients, and five WHO grade IV patients (GBM, IDHwt). Thus, the magnitude of FPIA uptake is not due to mutation status per se but likely the combination of factors that determine glioma grade, with the transformation of grade II to grade III in this small cohort implied for SUVmax ≥ 1.2 or TBRmax ≥ 2.4. Against the backdrop of a radiotracer with non-zero Ki, we speculate that TBRmean signifies TBR across the entire lesion, and for LGG, this could include many regions that are not transforming admixed with transforming regions and healthy brain tissue, with TBRmax representing maximum transformation within the region of interest. A counterargument is that noisy voxels could contribute to the TBRmean, given the low absolute value, and this fact should be examined in future studies. Further studies show that FPIA uptake is also high in brain metastasis (*Islam *et al*., unpublished*), which supports the notion of malignancy-related uptake. Other MRI methods that examine perfusion, blood volume, diffusion and tumour spectra have been investigated for malignancy prediction [44]. For example, v_e_ (DCE-MRI-derived extravascular-and-extracellular volume fraction; proposed cellularity marker) was higher in GBM compared to grades II and III tumours, consistent with v_e_ reported to be predictive of progression-free and overall survival in HGG patients [6], and DCE-MRI enhancing variables, in general, have been shown to been shown to be different in low- and high-grade gliomas [45]. We did not have sufficient patient numbers to comment on DCE variables and CBV in grade III patients with and without 1p/19q del, and this will be a future objective. ADC in HGG and IDHwt gliomas were lower than in LGG and IDHmu gliomas. For hybrid PET/MRI, the combination of FPIA and v_e_ was not selected by the penalised regression method, which instead suggested that combinations of FPIA PET and perfusion variables may be worthy of investigation. Further investigation of these variables and methodology in a larger patient population is warranted. Another area that needs further work is the use of FPIA PET to support volume delineation, as seen in discordance PET/MRI data in Fig. 2A and Supplementary Figs. S3 and S4. Validation of tumour extent by FPIA will transform the use of imaging to guide radiotherapy volume delineation as well as efficient surgical planning.

In conclusion, FPIA PET shows uptake in glioma, increasing in the order LGG (WHO grade II) < (WHO grade III) < HGG (WHO grade IV). The combined use of FPIA PET and v_e_ or perfusion variables in a PET/MRI context appear worthy of investigation in future studies.

### Supplementary Information

Below is the link to the electronic supplementary material.

Supplementary file1 (PPTX 4583 kb) **Supplementary Fig. S1: Multi-parametric FPIA PET/MRI image data for all acquisitions.** Axial PET and MRI images for grade II (PT01 and PT11), grade III (PT03, PT05, PT07) and grade IV (PT02, PT04, PT08, PT09, PT10) glioma patients including K1, Ki, Kep, Ve, Vp, taui, CBF, CBVlc, MTT and TTP. **Supplementary Fig. S2. PET whole-blood correction.** Standardized uptake value for last 5 time frames (SUV) and blood pool corrected SUV (SUVc) maps in a representative patients. Since the quantification of the local uptake of ^18^F-FPIA in brain tumours can be biased by the uptake of the radiotracer in the blood, for qualitative image interpretation, a voxel-wise SUV whole blood correction was performed as follows: $SUVc=SUV-nSUVwb$ where *SUVc* is the corrected SUV and *nSUVwb* is the normalised SUV evaluated in the whole blood (superior sagittal sinus). SUV quantification and correction were performed using an in-house software written in Matlab. **Supplementary Fig. S3.Image congruence for patients whose data are not shown in Figure 2.A.** lesion mask outlined on the post- contrast T1 MRI image (red) and on the SUV_40_ and SUV_30_ (green). **Supplementary Fig. S4. Relationship between diffusion, perfusion, and SUV**. Diffusion weighted MRI apparent diffusion coefficient (ADC), arterial spin labelling cerebral blood flow (CBF) and FPIA standardized uptake value (SUV) for grade II, III and IV lesions and contralateral white matter. **Supplementary Fig. S5.Plasma carnitine levels in individual patients at the time of PET scan**. **A.** Acyl carnitine measurements in the 10 patients. There was no restriction on food intake prior to scanning. Acetyl carnitine (C2, short chain fatty acid), (C3–C5, sum of short chain carboxylic acids C3, C4, C5; without C2), free carnitine and total carnitine are shown. **B.** Heatmap of Acyl carnitine measurement in the 10 patients. Acetyl carnitine (C2), short chain fatty acids (C3–C5), medium chain fatty acids (C6–C8), Long chain fatty acids (C10, C12, C14, C16, and C18). **Supplementary Fig. S6.** **Immunohistochemistry of enzymes involved in transport and esterification of SCFAs with a focus on carnitine species determined from a cohort of archival tissue independent of that employed in the imaging study.** Paraffin embedded tumour tissues (8 LGG, 25 HGG) were obtained from Imperial College Tissue Bank for analysis as described in Methods. Summary data for Ki-67 and five enzymes involved in SCFA transport, carnitine esterification and transport are indicated. **Supplementary Figure S7**>**.** **Bioinformatics assessment of the potential relevance of SLC25A20**. mRNA expression of **A.** the ubiquitous SLC16A1 (MCT1) and SLC25A20. **B. **Survival analysis of the combined LGG and GBM dataset. Data were analysed as reported in the methods section. **Supplementary Table S1.** **Imaging variables from PET/MRI study**.

## Data Availability

As image data cannot be fully anonymised, they cannot be shared in a public repository. The datasets generated during and/or analysed during the current study are available from the corresponding author upon reasonable request.
