# Supplementary material for: Feasibility of [18F]fluoropivalate hybrid PET/MRI for imaging lower and higher grade glioma: a prospective first-in-patient pilot study
Source: Eur J Nucl Med Mol Imaging. 2023 Jul 25;50(13):3982–95. doi: 10.1007/s00259-023-06330-0 (PMC10611885; doi:10.1007/s00259-023-06330-0)
Supplement: Supplementary file 1 — Supplementary file1 (PPTX 4583 kb) Supplementary Fig. S1: Multi-parametric FPIA PET/MRI image data for all acquisitions. Axial PET and MRI images for grade II (PT01 and PT11), grade III (PT03, PT05, PT07) and grade IV (PT02, PT04, PT08, PT09, PT10) glioma patients including K1, Ki, Kep, Ve, Vp, taui, CBF, CBVlc, MTT and TTP. Supplementary Fig. S2. PET whole-blood correction. Standardized uptake value for last 5 time frames (SUV) and blood pool corrected SUV (SUVc) maps in a representative patients. Since the quantification of the local uptake of 18F-FPIA in brain tumours can be biased by the uptake of the radiotracer in the blood, for qualitative image interpretation, a voxel-wise SUV whole blood correction was performed as follows: $SUVc=SUV-nSUVwb$SUVc=SUV-nSUVwb where SUVc is the corrected SUV and nSUVwb is the normalised SUV evaluated in the whole blood (superior sagittal sinus). SUV quantification and correction were performed using an in-house software written in Matlab. Supplementary Fig. S3.Image congruence for patients whose data are not shown in Figure 2.A. lesion mask outlined on the post- contrast T1 MRI image (red) and on the SUV40 and SUV30 (green). Supplementary Fig. S4. Relationship between diffusion, perfusion, and SUV. Diffusion weighted MRI apparent diffusion coefficient (ADC), arterial spin labelling cerebral blood flow (CBF) and FPIA standardized uptake value (SUV) for grade II, III and IV lesions and contralateral white matter. Supplementary Fig. S5.Plasma carnitine levels in individual patients at the time of PET scan. A. Acyl carnitine measurements in the 10 patients. There was no restriction on food intake prior to scanning. Acetyl carnitine (C2, short chain fatty acid), (C3–C5, sum of short chain carb [file 259_2023_6330_MOESM1_ESM.pptx]

## Slide 1
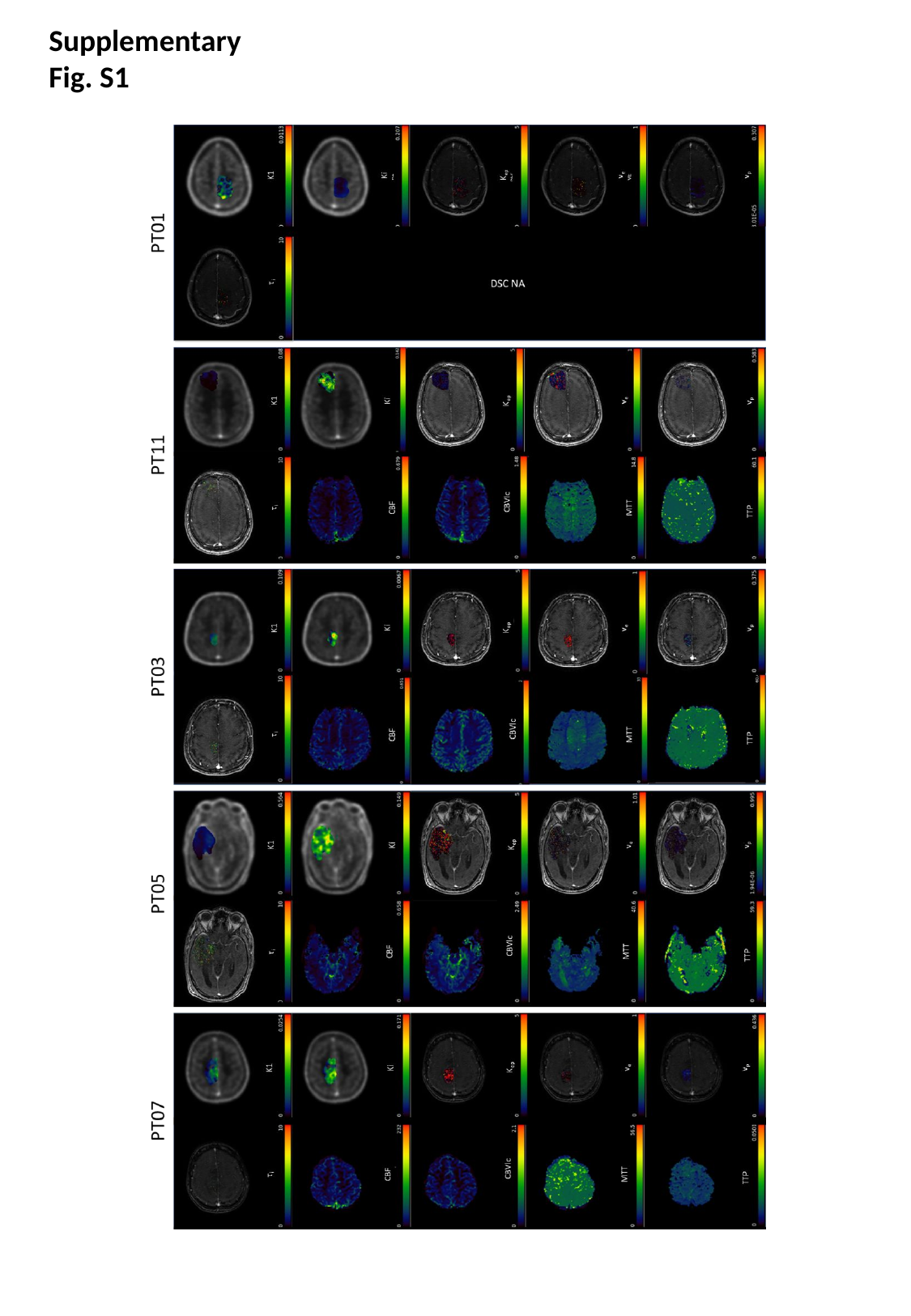

Supplementary Fig. S1

## Slide 2
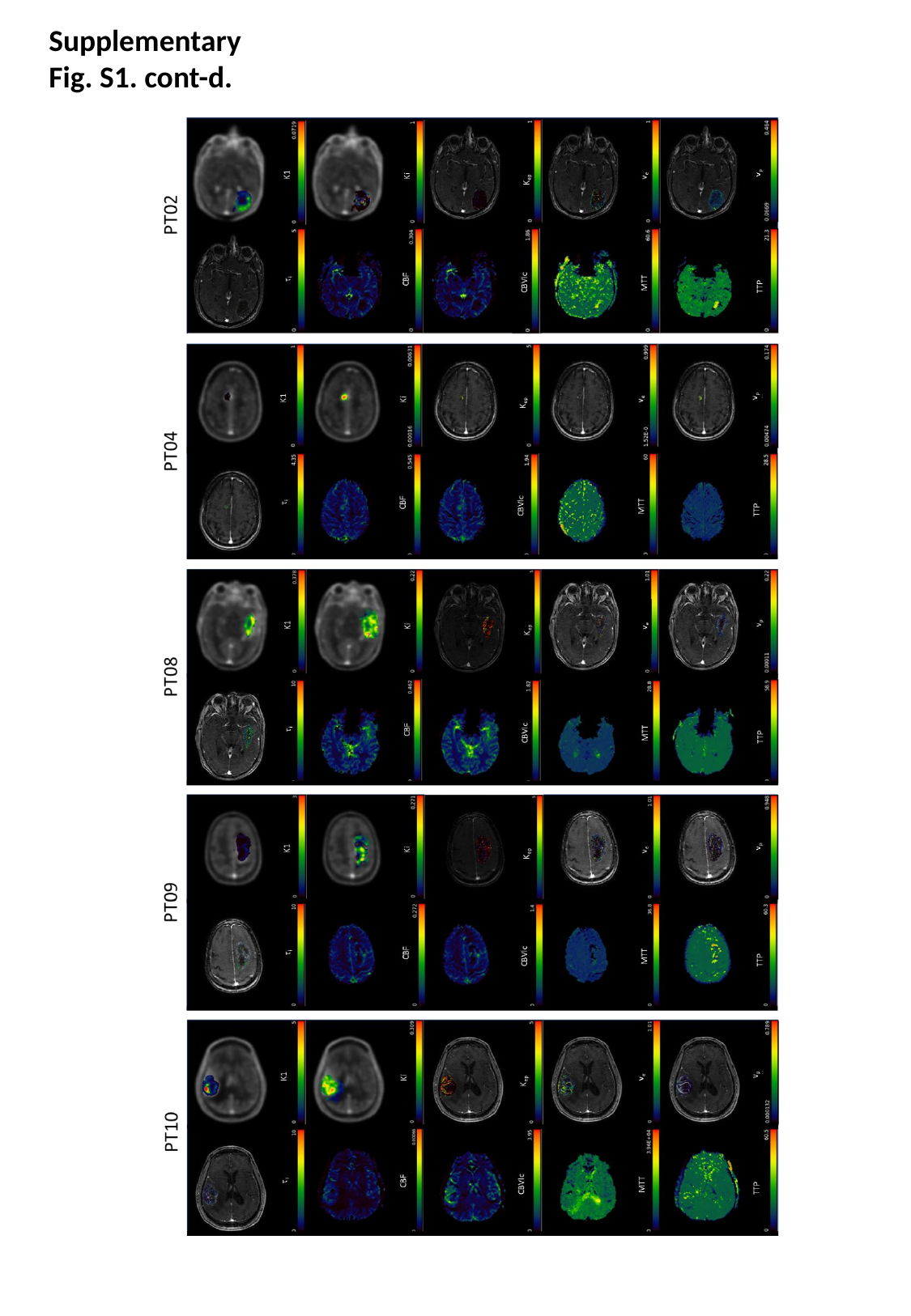

Supplementary Fig. S1. cont-d.

## Slide 3
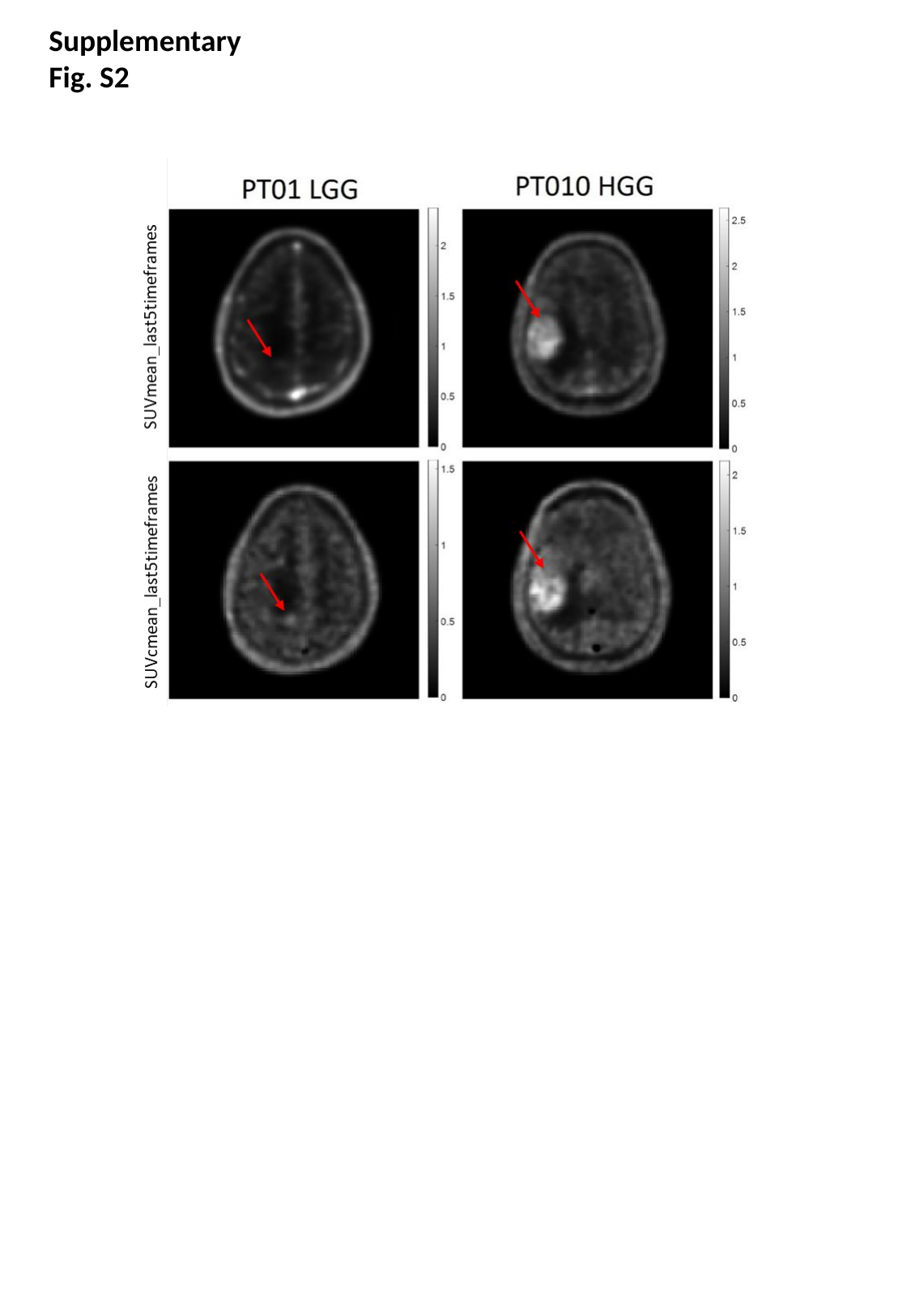

Supplementary Fig. S2

## Slide 4
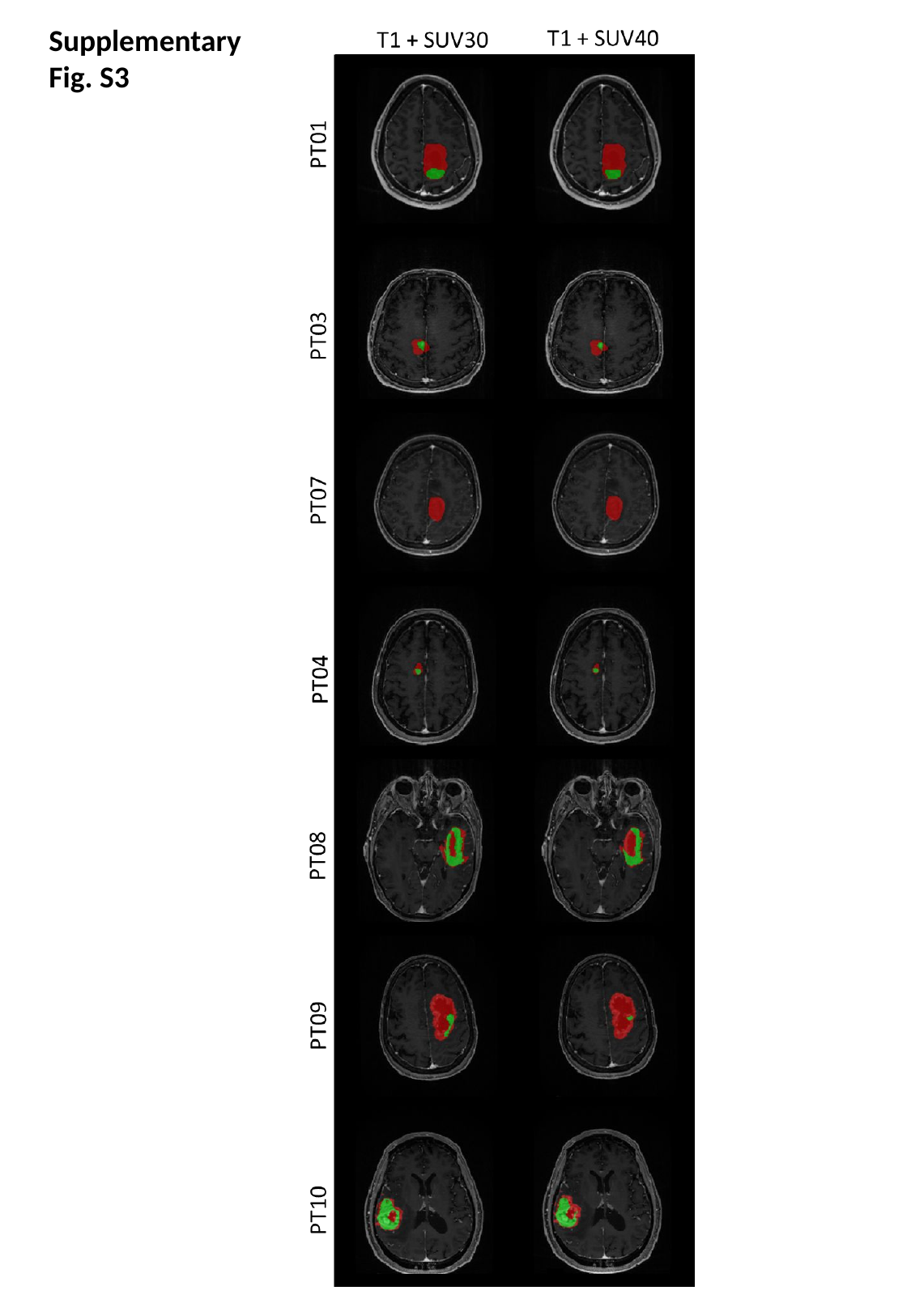

Supplementary Fig. S3

## Slide 5
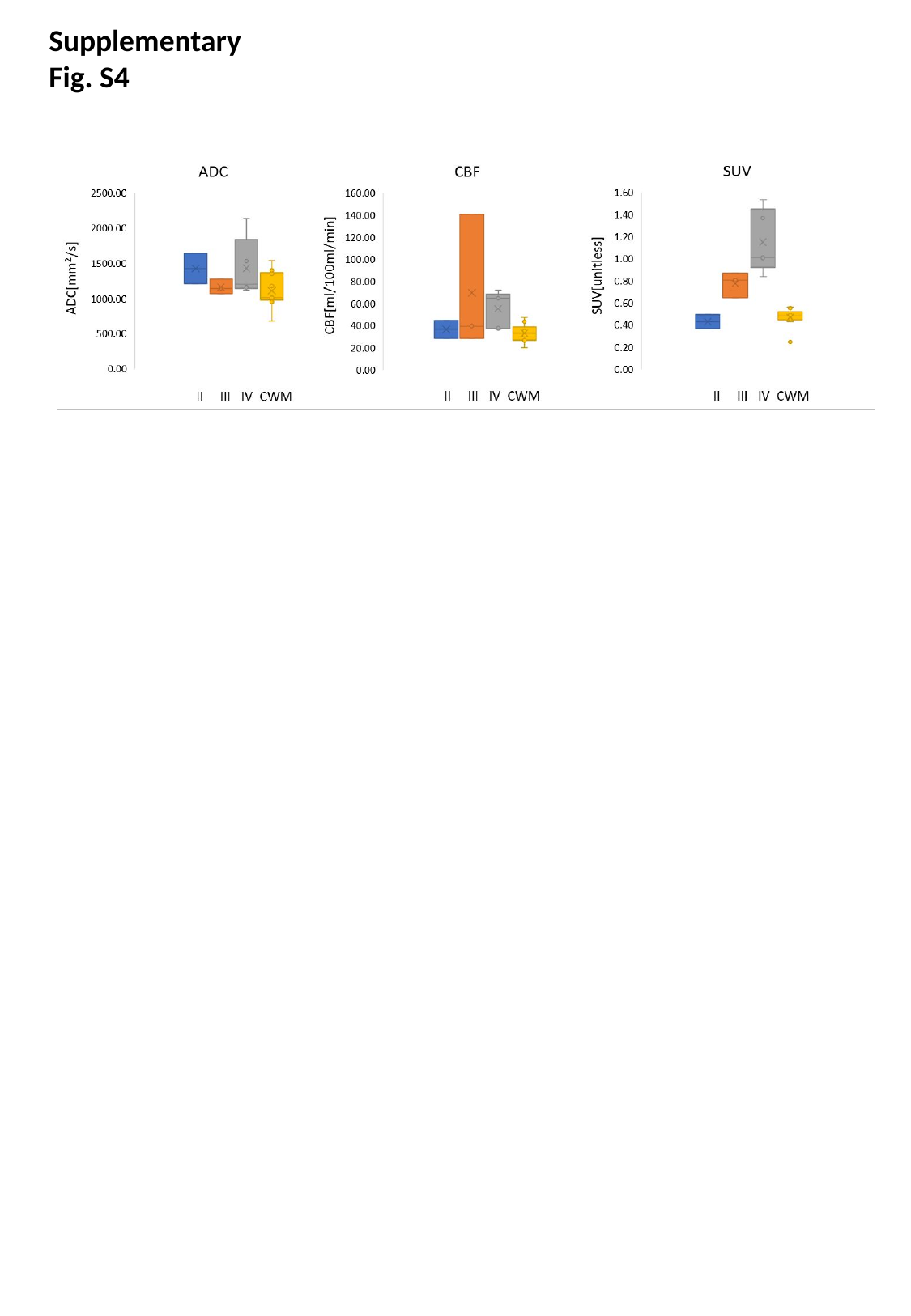

Supplementary Fig. S4

## Slide 6
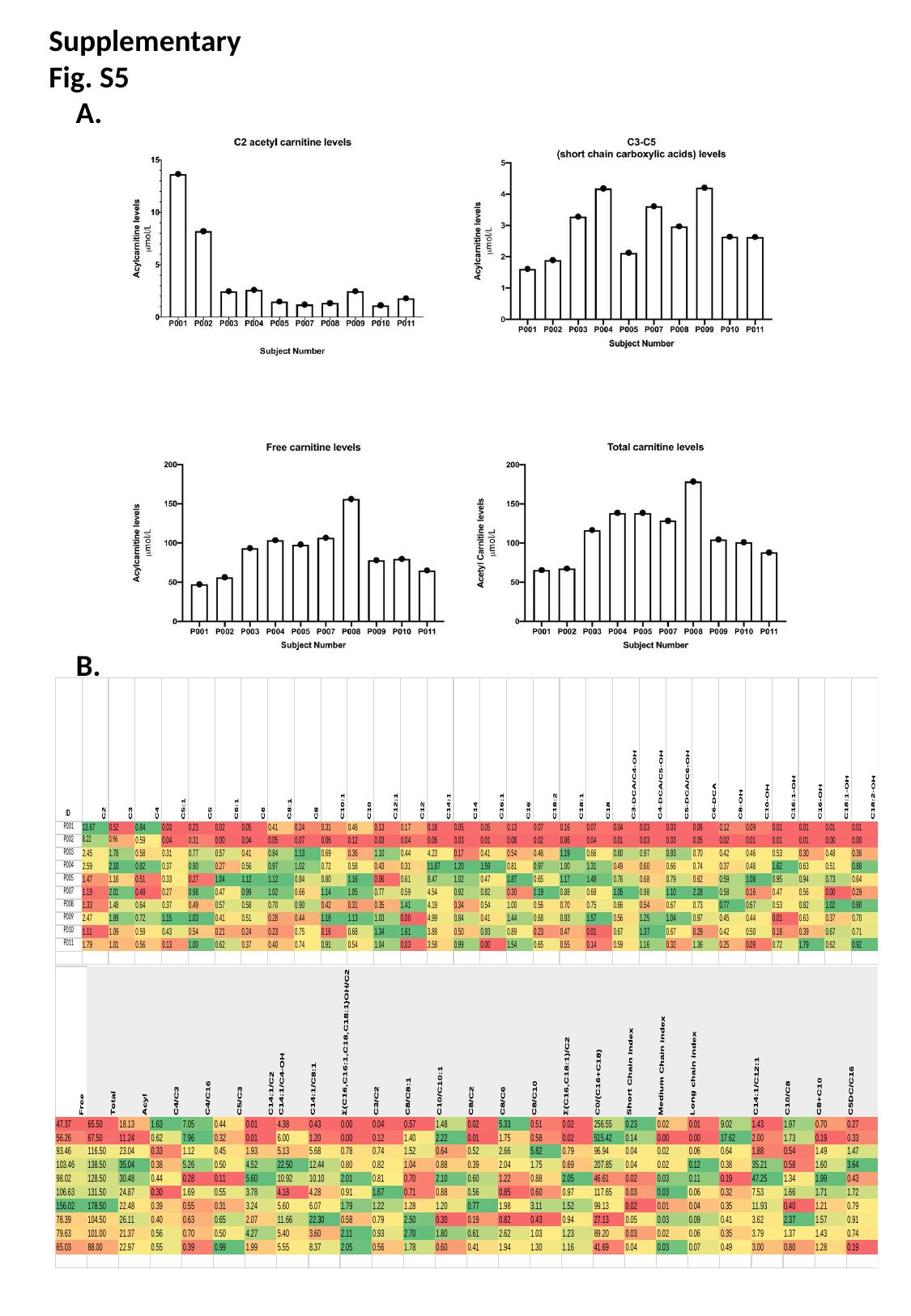

Supplementary Fig. S5
A.
B.

## Slide 7
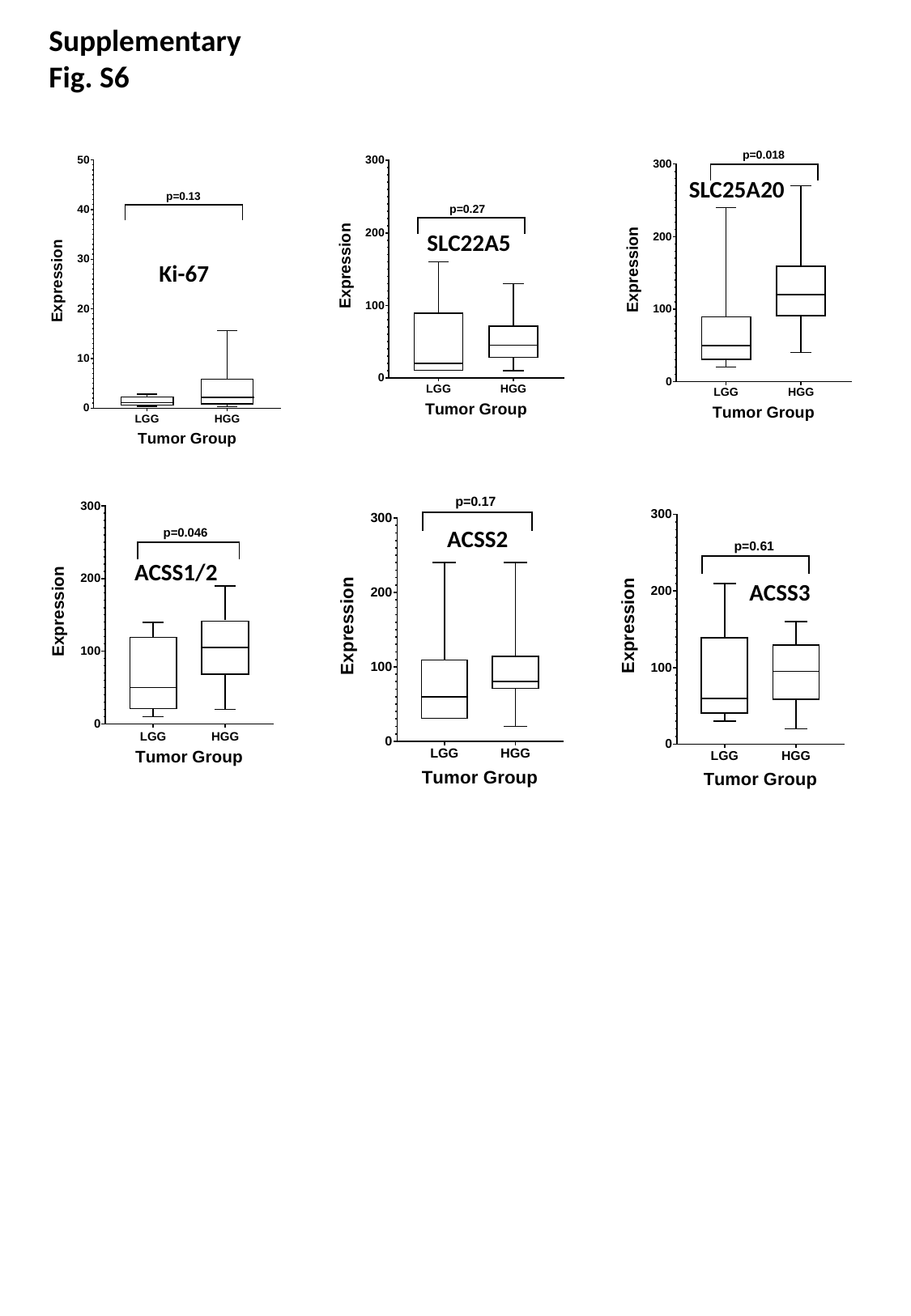

Supplementary Fig. S6
SLC25A20
SLC22A5
Ki-67
ACSS2
ACSS1/2
ACSS3

## Slide 8
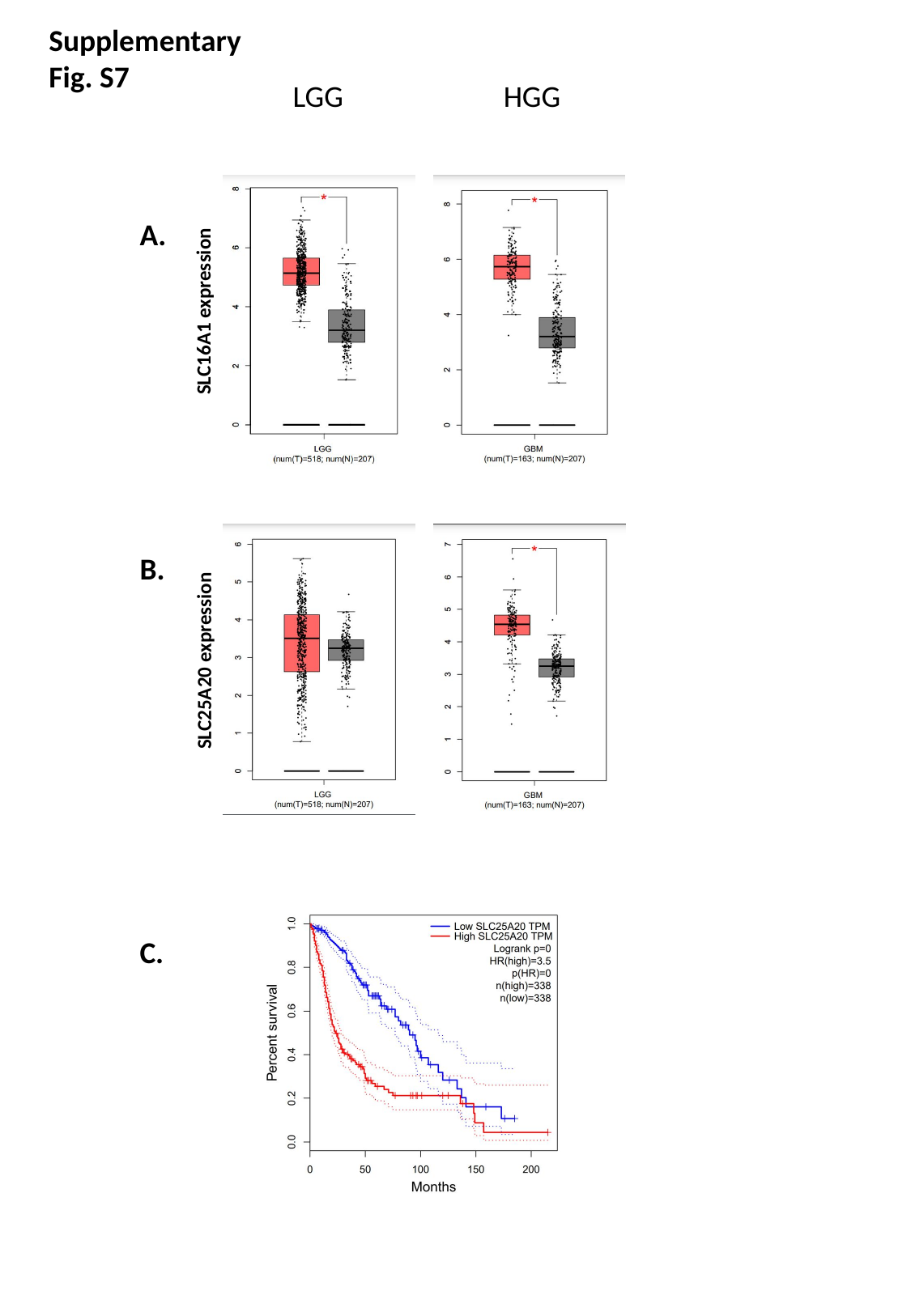

Supplementary Fig. S7
LGG
HGG
A.
SLC16A1 expression
B.
SLC25A20 expression
C.

## Slide 9
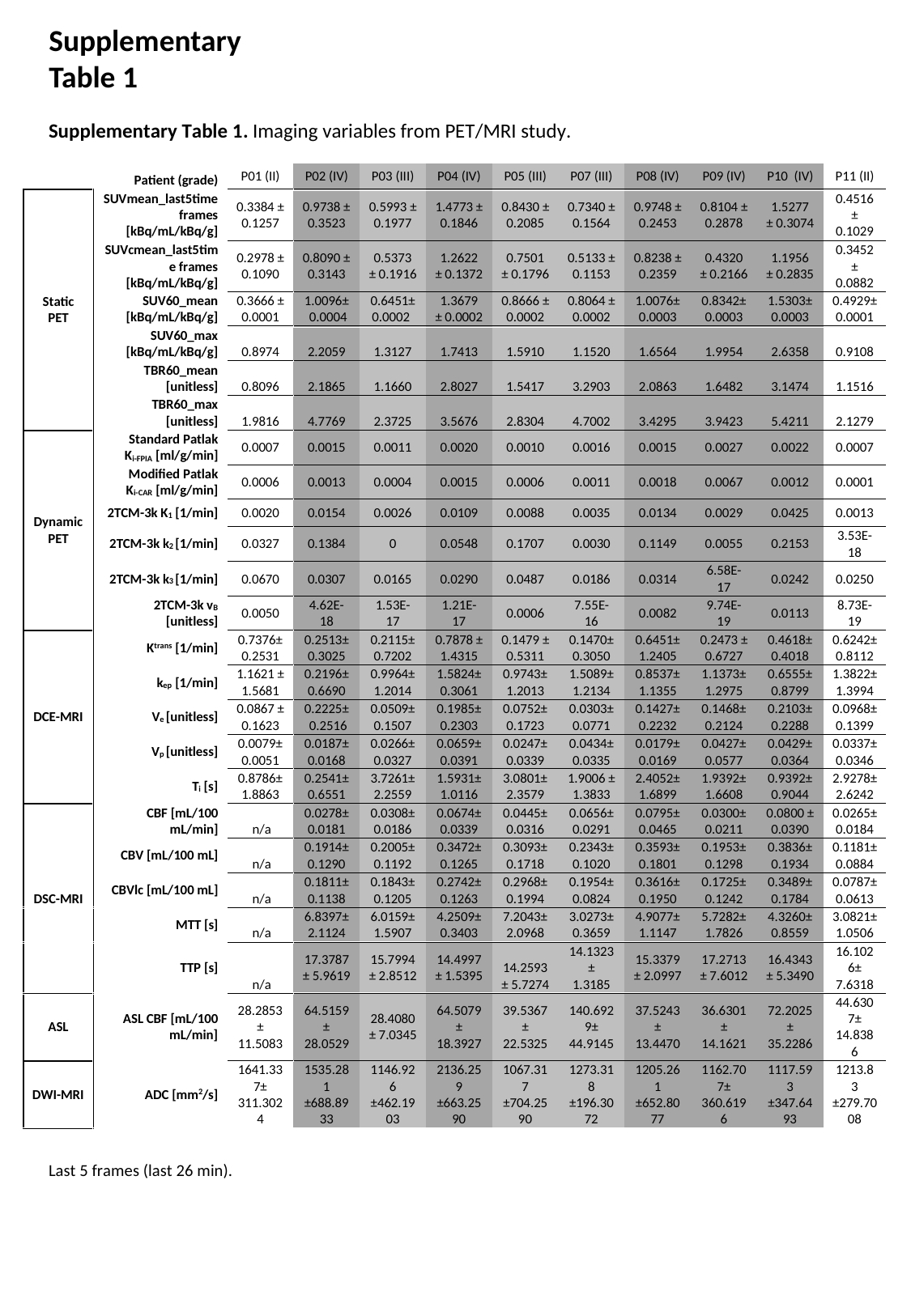

Supplementary Table 1
